# Diagnosis and prognostic value of C-X-C motif chemokine ligand 1 in colon adenocarcinoma based on The Cancer Genome Atlas and Guangxi cohort

**DOI:** 10.7150/jca.51524

**Published:** 2021-07-25

**Authors:** Yi-Zhen Gong, Hui Ma, Guo-Tian Ruan, Li-Chen Zhu, Xi-Wen Liao, Shuai Wang, Ling Yan, Wei Huang, Ke-Tuan Huang, Hailun Xie, Guang-Zhi Zhu, Xiang-Kun Wang, Cun Liao, Feng Gao

**Affiliations:** 1 Department of Colorectal and Anal Surgery, The First Affiliated Hospital of Guangxi Medical University, Nanning, Guangxi Zhuang Autonomous Region, People's Republic of China.; 2 Department of Immunology, School of Preclinical Medicine, Guangxi Medical University, Nanning, Guangxi Zhuang Autonomous Region, People's Republic of China.; 3 Department of Hepatobiliary Surgery, The First Affiliated Hospital of Guangxi Medical University, Nanning, Guangxi Zhuang Autonomous Region, People's Republic of China.

**Keywords:** *CXCL1*, COAD, Diagnosis, Prognosis, Biomarker, GSEA

## Abstract

**Objective:** The objective was to identify and validate C-X-C motif chemokine ligand 1(*CXCL1*) for diagnosis and prognosis in colon adenocarcinoma (COAD).

**Methods:** Our current study had enrolled one The Cancer Genome Atlas (TCGA) cohort and two Guangxi cohorts to identify and verify the diagnostic and prognostic values of *CXCL1* in COAD. Functional enrichment was performed by gene set enrichment analysis (GSEA).

**Results:** In TCGA cohort, the expression of *CXCL1* was significantly up-regulated in tumor tissues and decreased as the tumor stage developed. The receiver operating characteristic (ROC) curve showed that *CXCL1* had a high diagnostic value for COAD. The result of Kaplan-Meier survival analysis showed that *CXCL1* gene expression (*P*=0.045) was significantly correlated with overall survival (OS) of COAD. Results of Guangxi cohort also verified the diagnostic value of *CXCL1* in COAD, and sub-group survival analyses also suggested that patients with high *CXCL1* expression were related to a favorable OS (Corrected *P*=0.005). GSEA revealed that *CXCL1* high expression phenotype was related to cytokine activity, cell apoptosis, *P53* regulation pathway, and regulation of autophagy in COAD.

**Conclusions:** In this study, we found that *CXCL1* gene might be a potential diagnostic biomarker for COAD, and might serve as a prognostic biomarker for specific subgroup of COAD.

## Introduction

Colorectal cancer (CRC) ranks as one of those diseases of the highest morbidity and mortality in the world 1. The early treatment of CRC had a good prognosis, and the survival rate of patients with early cancer was about five times higher than that of patients with advanced cancer 2. Colonoscopy remained the gold standard for CRC diagnosis, but the procedure was invasive, expensive, and had low patients' acceptance. Serum carcinoembryonic antigen (CEA) is a tumor marker and is more meaningful for CRC diagnosis and postoperative monitoring. However, serum CEA positive rate in CRC patients was less than 50% in some clinical trials 3-5. Therefore, it was necessary to identify better biomarker to improve the effectiveness of early diagnosis of CRC and prognosis prediction of the patients.

CXC motif chemokine ligand 1 (*CXCL1*), also known as the GRO1 oncogene, is a small cytokine of the CXC chemokine family 6. It is expressed by macrophages, neutrophils, and epithelial cells and has neutrophil chemoattractant activity 6-8. *CXCL1* was taken part in the processes of angiogenesis, arteriogenesis, inflammation, wound healing, and tumorigenesis 9, 10. This chemokine triggered its above actions by signaling from the chemokine receptor CXCR2 10. Previous researches had discovered that *CXCL1* was markedly upregulated in CRC cancer tissues 11, 12, and the overexpress of *CXCL1* was connected to the poor prognosis of CRC stage III 13.

More than 60% of CRC occurred in the colon. Global cancer statistics showed 1,096,601 new colon cancer cases and 551,269 deaths in 2018, accounting for about 6% of all tumors 1. The cause of colon cancer is not the same as that of rectal cancer 1, 14, so their pathogenesis might also be different. Majority of the pathological type of colon cancer is colon adenocarcinoma (COAD). Previous studies had not systematically reported the diagnostic and prognostic value of *CXCL1* in COAD. In this study, we first explored the diagnostic and prognostic values of *CXCL1* gene mRNA expression in COAD applying the Cancer Genome Atlas (TCGA) database, and then validated the TCGA results with the cohort of the First Affiliated Hospital of Guangxi Medical University.

## Materials and Methods

### Expression of *CXCL1* in COAD and normal tissues

Human Protein Atlas (HPA: https://www.proteinatlas.org, accessed December 27, 2018) is used to reveal the distribution of CXCL1 in normal human tissues 15. Gene Expression Profiling Interactive Analysis (GEPIA, http://gepia.cancer-pku.cn/detail.php?gene=cxcl1, accessed February 17, 2019) was used to explore the distribution of *CXCL1* between COAD and normal colon tissues 16.

### RNA sequencing data in TCGA

RNA sequencing dataset and patient parameters of COAD was got from TCGA (https://cancergenome.nih.gov, Accessed time: November 27, 2018) 17, 18. We compared the expression of *CXCL1* in tumor and paracancerous tissues of COAD patients to evaluate its diagnostic value, high- and low-expression CXCL1 phenotypes of COAD for survival analysis were grouped according to median value.

### Real-time RT-PCR (RT-qPCR) of *CXCL1* expressions in COAD

#### Patient tissue samples

From April to June 2018, we continuously collected tumor and paracancerous tissues from the surgery of the patients with COAD in the Department of Colorectal and Anal Surgery, the First Affiliated Hospital of Guangxi Medical University (Nanning, Guangxi). The patient's tissue was soaked in RNA store reagent immediately after surgery and subsequently frozen in a -80 ° C refrigerator. These patients were those who had no radiation or chemotherapy before surgery and their postoperative pathological diagnosis was COAD. In the Guangxi cohort, we only collected tissues from patients who had not received preoperative chemoradiotherapy and who were pathologically confirmed to have COAD after surgery. All patients in this study signed informed consent, and the Ethics Committee of the First Affiliated Hospital of Guangxi Medical University approved the experimental protocol [Ethics no.:2019(KY-E-001)].

#### RNA extraction and RT-qPCR

First, we extracted the total RNA from tissues via the TRIzol reagent (15596026, Invitrogen). Then, we applied the PrimeScript™ RT Reagent Kit with gDNA Eraser (RR047A, Takara) to synthesize the total RNA into first-strand cDNA. After that, the expression of *CXCL1* was normalized to GAPDH expression. At the same time, quantitative real-time PCR (qPCR) was conducted via the FastStart Universal SYBR Green Master (ROX) (Roche) in the Applied Biosystems Quantsudio TM Real-PCR System (Q6). All the above experiments were carried out according to the instructions. The relative gene expression level was performed according to 2^‑ ∆∆^ Ct 19, 20.

The primer sequences were as follows:

*CXCL1*-forward: 5'-CAAACCGAAGTCATAGCCACA-3'

*CXCL1*-reverse: 5'-CTCCTAAGCGATGCTCAAACA-3'

*GAPDH*-forward: 5'-GTCAGCCGCATCTTCTTT-3'

*GAPDH*-reverse: 5'-CGCCCAATACGACCAAAT-3'

### Immunohistochemistry (IHC) of *CXCL1* expressions in COAD

#### Patient tissue samples

We retrospectively collected tumor and paracancerous tissues wax blocks from patients who underwent colonic tumor resection in the First Affiliated Hospital of Guangxi Medical University from 2012 to 2013. The patients did not have any other known tumors. No radiotherapy or chemotherapy had been performed before surgery. The pathological diagnosis was COAD, and the tumors were identified and categorized according to the American Joint Committee on Cancer (AJCC) tumor node metastasis (TNM) staging system (8th edition, 2017) 21. We routinely collected clinical parameters and survival dataset for these patients. Inclusion criteria for COAD patients were described above. All patients in this study signed informed consent, and the Ethics Committee of the First Affiliated Hospital of Guangxi Medical University approved the experimental protocol [Ethics no.:2019(KY-E-001)].

#### Evaluation of IHC

We used the *CXCL1* antibody supplied by Signalway Antibody LLC, and the immunohistochemical staining reagents from Shanghai ChangDao Biotech Company, China. IHC procedure carried out in accordance with the manufacturer's instructions. Two pathologists respectively evaluated the percentage of positive cells according to the following norm: 0 (0%); 1 (1-25%); 2 (26-50%); 3 (51-75%); and 4 (76- 100%). According to the intensity of staining, the staining results were divided into four levels: negative, weak, moderate and strong, and give four corresponding scores of 0, 1, 2, and 3, respectively. We multiply the percentage and staining intensity score to get the final IHC score. The results of these two independent pathologists were calculated and got the average score. When the scores were over two, the positive staining results were confirmed 22.

#### Gene set enrichment analysis (GSEA)

We divided the TCGA patients into two groups, as high one and low on, based on the expression of *CXCL1*. Then we applied GSEA (http://software.broadinstitute.org/gsea/index.jsp, accessed December 24, 2018) v3.0 to investigate the prognosis molecular mechanism of *CXCL1* in patients with COAD by enriching metabolic pathways and biological processes 23. The reference gene sets of GSEA were obtained from Molecular Signatures Database (MSIGDB): c5 (GO gene sets: bp. V6.2.symbols.gmt, cc.v6.2.symbols.gmt, and mf. v6.2.symbols.gmt) and c2 (KEGG gene sets: c2.cp.kegg.v6.2.symbols.gmt), respectively. Nominal *P* less than 0.05 and false discovery rate (FDR) less than 0.25 were considered statistically significant.

### Statistical Analysis

We conducted a t-test to assess the *CXCL1* expression between tumor and paracancerous tissues. Kaplan-Meier method was performed for survival analysis. We applied the Cox regression model to evaluate the hazard ratio (HR) and 95% confidence interval (CI). The FDR in GSEA was carried out according to the Benjamini-Hochberg procedure 24, 25. The drawing of the figures were performed by GraphPad Prism 7.0. *P*<0.05 was regarded as statistically significant. SPSS v.24.0 software (IBM, Chicago, IL, USA) was used for statistics.

## Results

### Expression of *CXCL1* in COAD and normal tissues

**Figure 1:**
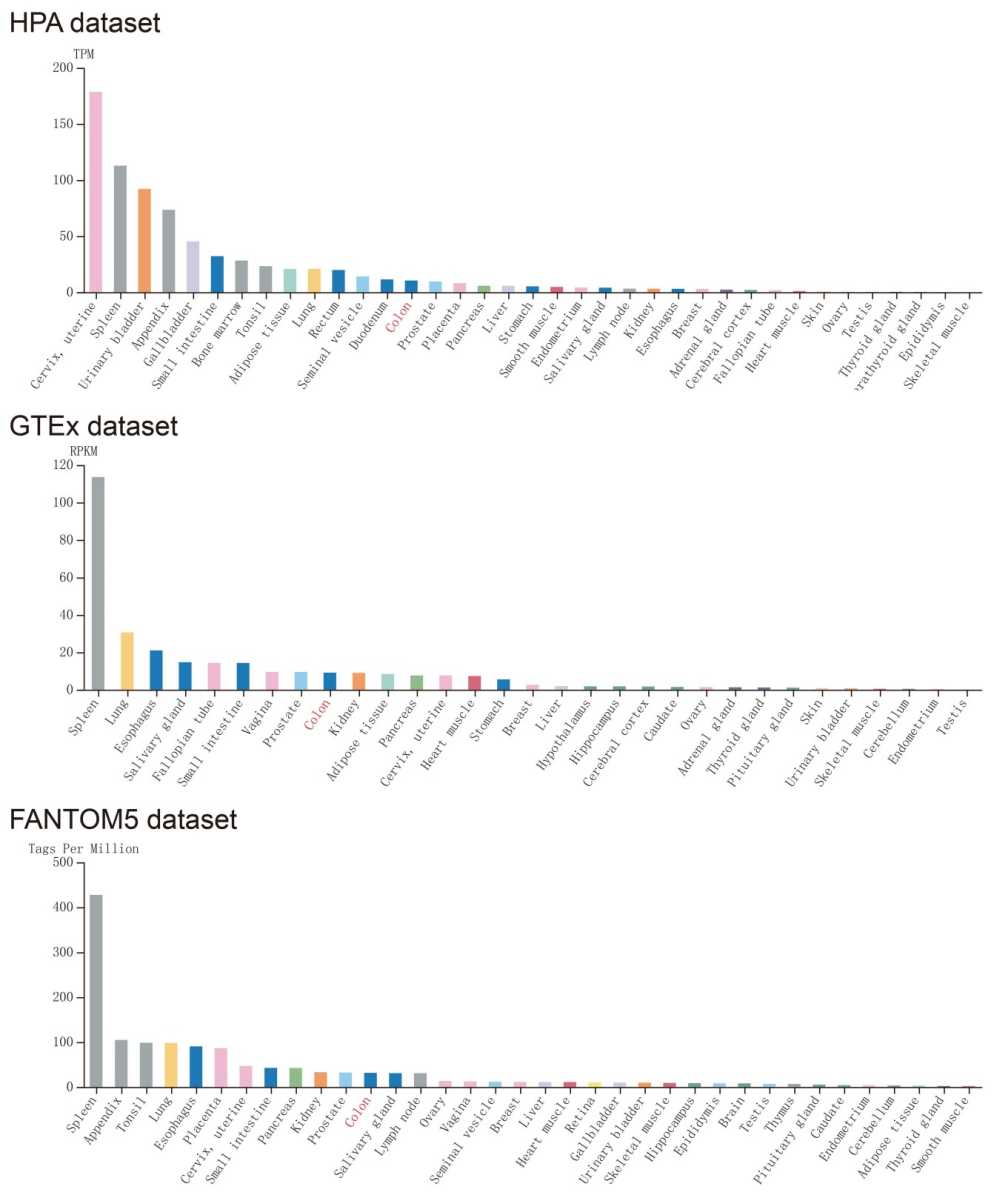
The expression level of *CXCL1* in normal human tissues. Notes: GTEx, Genotype-Tissue Expression; FANTOM5, the Functional Annotation of Mammalian Genomes 5.

**Figure 2:**
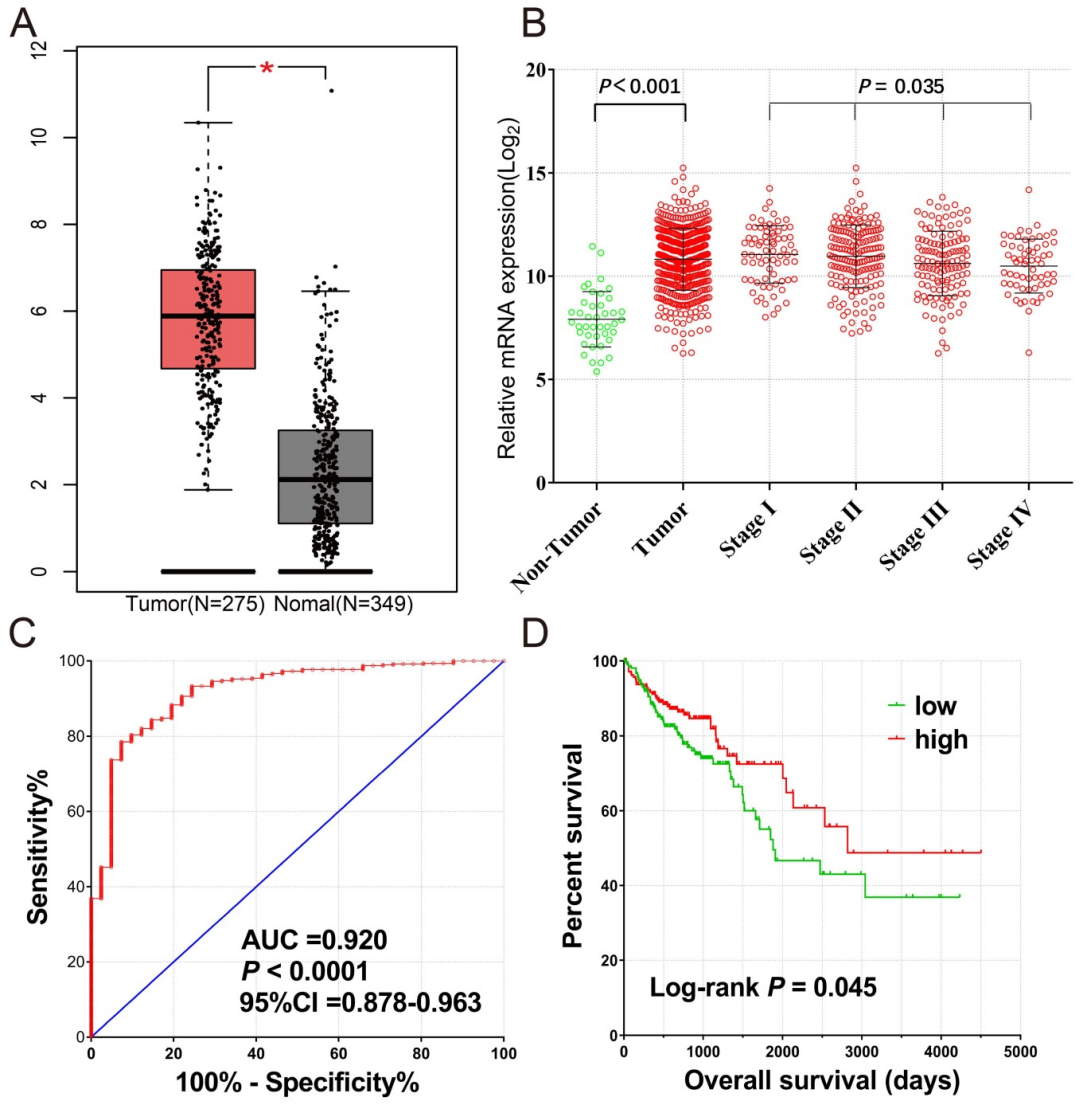
(A) The mRNA expression levels of *CXCL1* gene comparison in normal colon tissue and primary colon tumors based on TCGA and GTEx database; (B) The Scatter plot of *CXCL1* gene mRNA expression in tumor tissues and paracancerous non-tumor colon tissues of TCGA cohort, as well as those of different tumor stages; (C) The ROC curves of *CXCL1* gene mRNA expression in tumor tissues and paracancerous non-tumor colon tissues of TCGA cohort; (D) Kaplan-Meier curves for *CXCL1* gene in COAD of TCGA cohort.

Expression of *CXCL1* in normal human tissues was got from Human Protein Atlas, which was based on Functional Annotation of Mammalian Genomes 5 (FANTOM5), Genotype-Tissue Expression (GTEx), and HPA RNA-seq dataset (Fig. 1), *CXCL1* gene was highly expressed in normal human colon tissue. The expression of *CXCL1* gene in COAD tumor tissues was significantly higher than that in normal colon tissues (Fig. 2a).

### COAD data analysis in TCGA database

A total of 461 COAD patients were enrolled in the project. There were RNA sequencing data in 480 tumor and 41 paracancerous tissue samples from 456 patients. The expression of *CXCL1* was markedly up-regulated in tumor tissues, and it decreased as the tumor stage developed (Fig. 2b). The ROC curve (Fig. 2c) showed that *CXCL1* had a high accurately for COAD diagnosis [AUC(95% CI)=0.920(0.878-0.963)]. We excluded 5 patients without mRNA expression data, 2 patients without clinical data, 1 patient with postoperative survival time of “unknown”, and 15 patients with postoperative survival time of 0. Finally, 438 COAD patients with both survival data and genome-wide RNA sequencing data were included for survival analysis (Table 1). The results of the Kaplan-Meier survival analysis showed that the TNM stage (Log-rank* P*<0.0001) and *CXCL1* gene expression (*P*=0.045) were significantly correlated with overall survival (OS) of COAD (Fig 2d). However, the results of multivariate analysis showed that the OS of the *CXCL1* gene and COAD was not statistically significant in the correction of TNM staging of tumors (Corrected *P*=0.364, Corrected HR (95% CI) = 0.825 (0.544-1.250)).

### The mRNA expression of *CXCL1* in Guangxi COAD cohort

**Figure 3:**
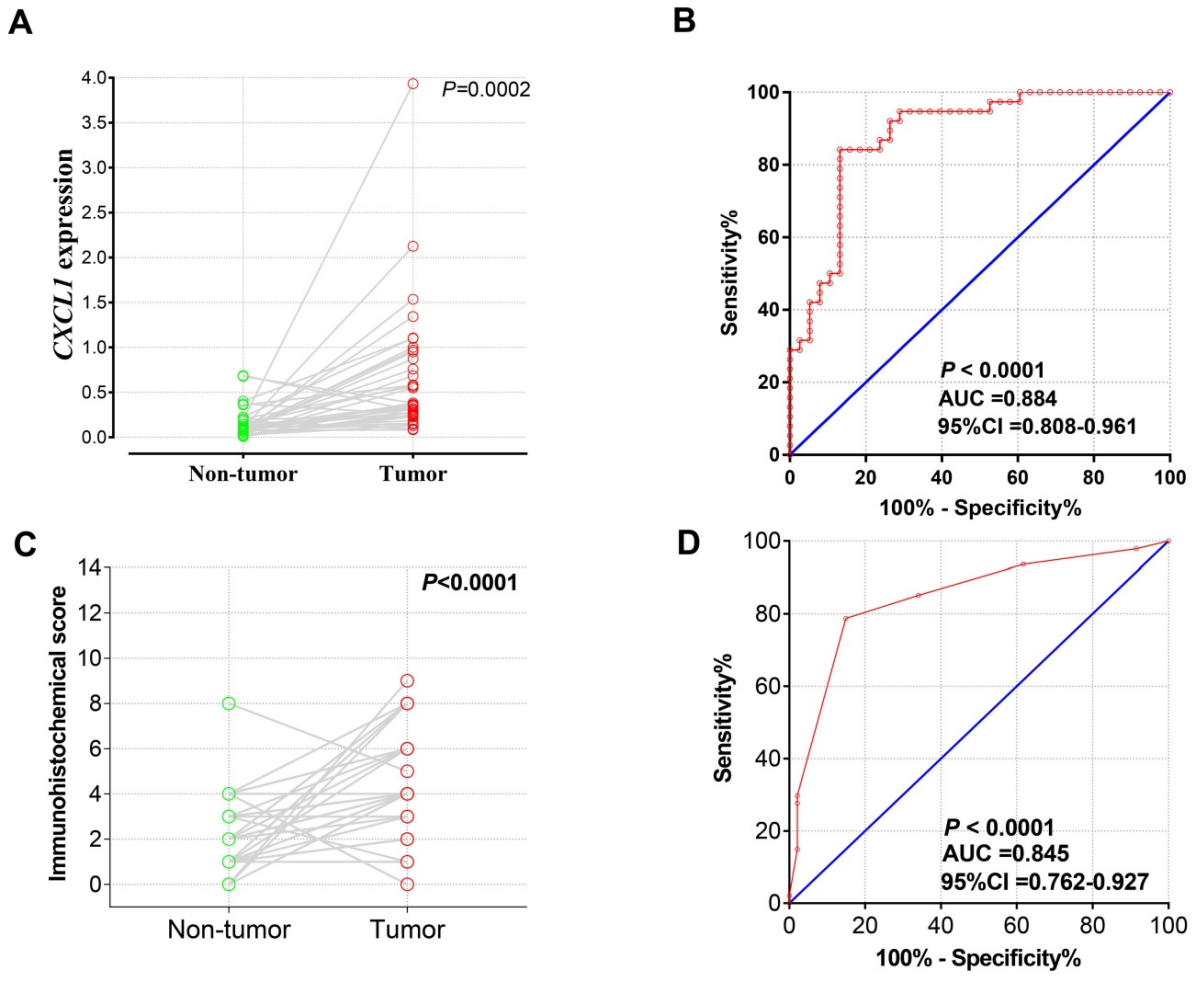
*CXCL1* mRNA expression in Guangxi Medical University COAD cohorts. Notes: The Scatter plot (A) and the ROC curves (B) of 38 pairs samples with RT-qPCR. The Scatter plot (C) and the ROC curves (D) of 47 pairs of samples with IHC.

A total of 38 patients with COAD were recruited into current study, with a median age of 61 years (ranged 35 to 85 years), 25 men and 13 women. The result of the pair-t test showed that *CXCL1* mRNA expression in COAD tumor tissues was markedly up-regulated than in paracancerous non-tumor colon tissues (Fig. 3a), and the diagnostic ROC curve (Fig. 3b) showed that *CXCL1* had a high accurately for COAD diagnosis (*P*<0.0001, AUC (95% Cl)=0.884(0.808-0.961) ).

### IHC expression of *CXCL1* in Guangxi COAD cohort

#### Basic characteristics of the study population

**Figure 4:**
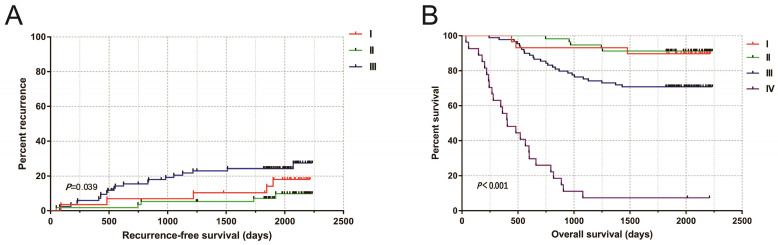
(A) Kaplan-Meier curves of TNM stage in COAD RFS; (B) Kaplan-Meier curves of TNM stage in COAD OS.

In this study, a total of 216 patients with COAD were retrospectively collected, 4 cases of tumor tissue wax mass could not be obtained, and 212 patients were included in the study (including 212 tumor tissues and 47 paracancerous non-tumor colon tissues). The median age was 59 years (ranged 17 to 87 years). The median follow-up time was 1934 days (ranged 36 to 2236 days). Ten people lost to follow-up. The tumor-free survival curves of COAD patients performed radical resection were shown in Fig 4a. The 5-year survival rate of TNM stage I and II patients was 90.7% that of stage III patients was 70.8%, and that of stage IV patients was 7.41% (Fig. 4b).

#### IHC results and clinicopathological factors

**Figure 5:**
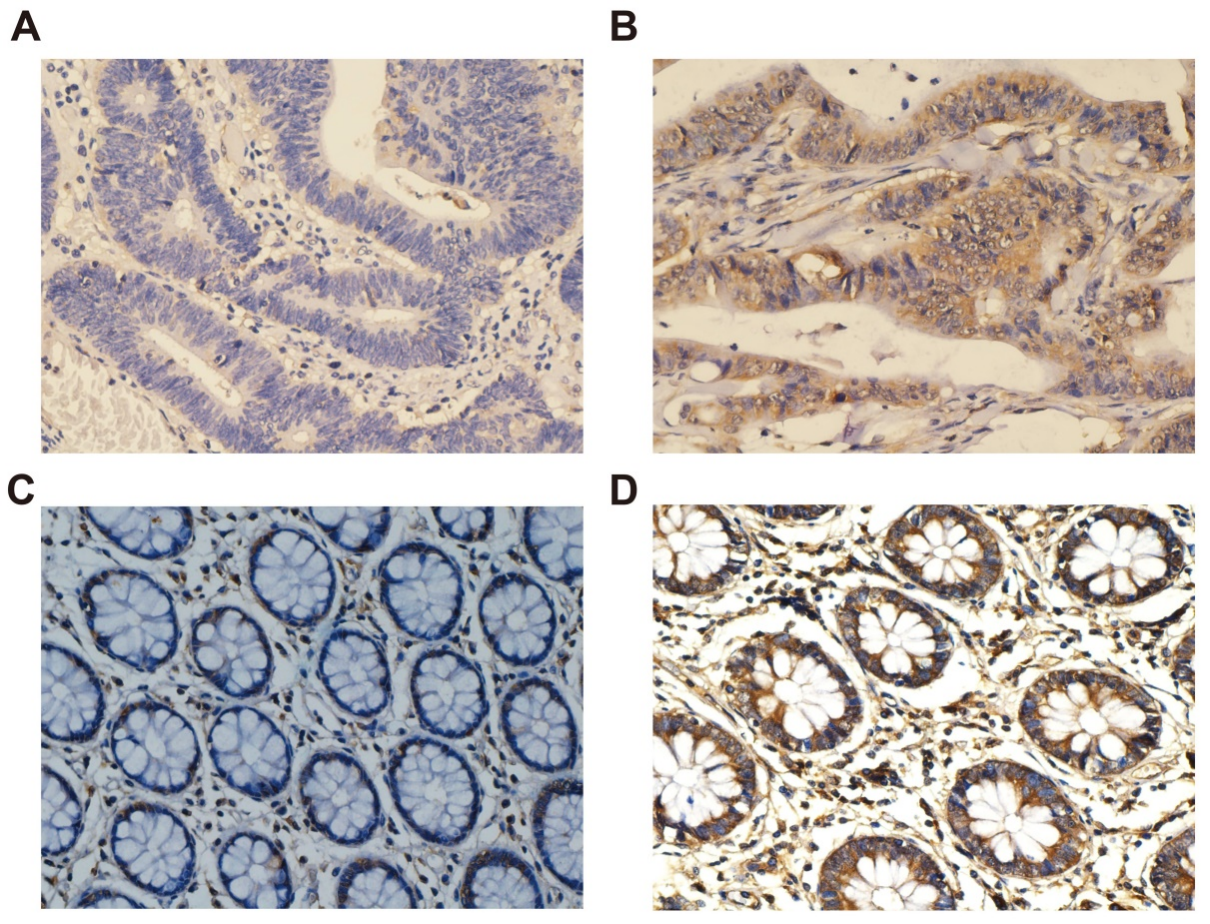
Immunohistochemical staining of *CXCL1* expression in COAD. Notes: *CXCL1* signaling was predominantly under the observation in the cytoplasm of colon cancer cells. (A) negative staining in tumor tissues; (B) positive expression in tumor tissues; (C) negative staining in paracancerous non-tumor colon tissues; (D) positive expression in paracancerous non-tumor colon tissues. Magnification, 400×.

The positive signal of *CXCL1* was the formation of diffuse brownish yellow or dark brown in the cytoplasm of the target cells (Fig. 5). The positive rate of *CXCL1* staining was 81.6% (173 / 212) in COAD patients and 34.0% (16 / 47) in paracancerous non-tumor colon tissues. We collected clinicopathological factors that might be relevant to prognosis to perform correlation analysis with *CXCL1*, the results showed that the expression of *CXCL1* protein in COAD patients was correlated with preoperative carcinoembryonic antigen (CEA) (Table 2).

#### Analysis of the diagnostic value of *CXCL1* Immunohistochemical staining

Paired t-test analysis showed that the immunohistochemical score of *CXCL1* in COAD carcinoma was considerably higher than that in paracancerous non-tumor colon tissues (Fig. 3c). At the same time, the results of the diagnostic ROC curve (Fig. 3d) revealed that *CXCL1* has a high accurately for COAD diagnosis (*P* < 0.0001, AUC= 0.845, 95% Cl ( 0.762 - 0.927).

#### Prognostic value of *CXCL1* immunohistochemical staining in COAD

**Figure 6:**
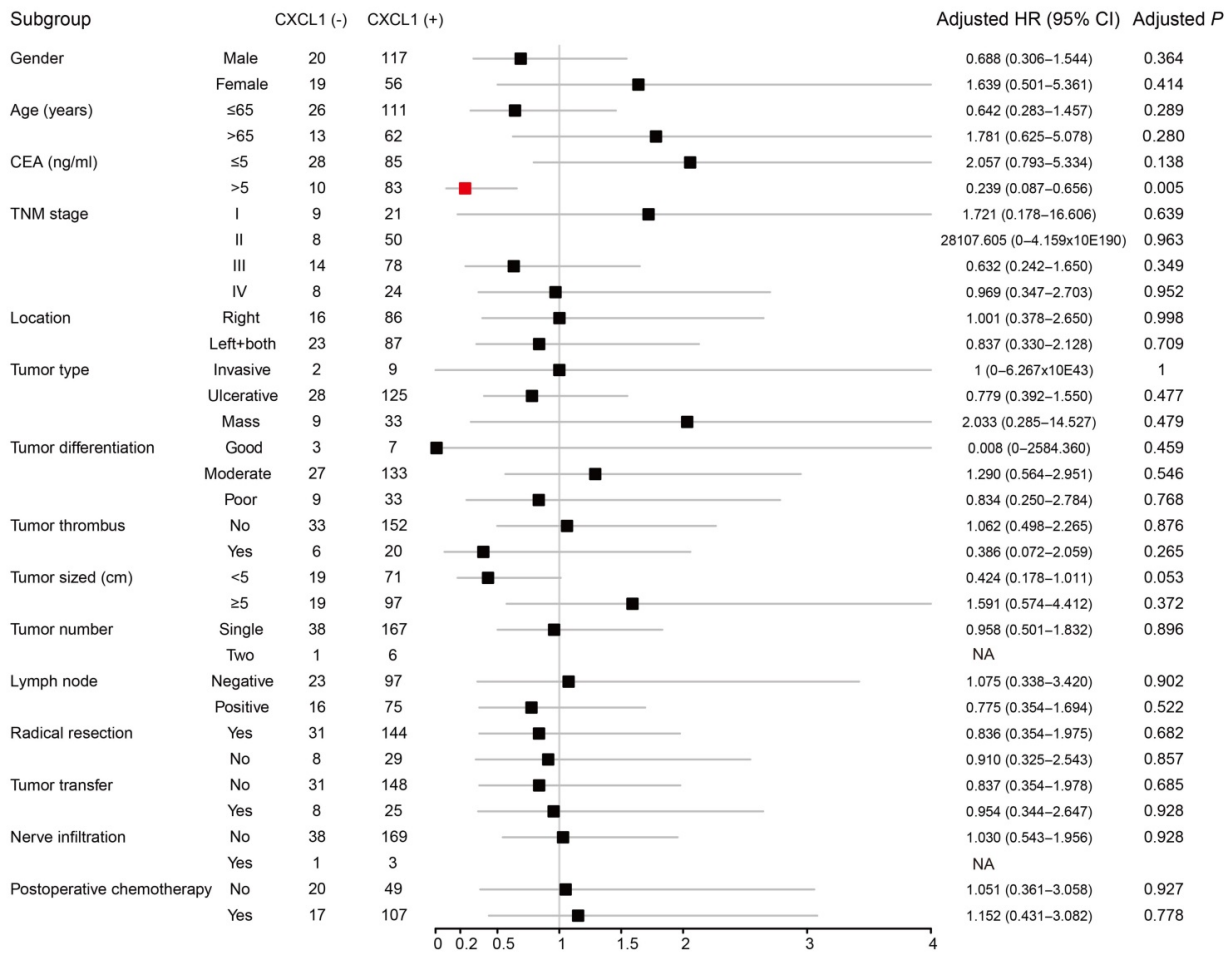
Forest plot of the stratified analysis of *CXCL1* with OS in different strata of characteristics in Guangxi cohort. Note: 6 patients without CEA information; 6 patients without general classification information; 1 patient without tumor thrombosis information; 6 patients without tumor size information; 1 patient without lymph node information; 1 patient without nerve infiltration information; 19 patients without postoperative chemotherapy information; NA, not obtained

We performed Kaplan-Meier analysis to compare clinicopathological factors and prognosis of COAD patients (Table 3), the results showed that the recurrence-free survival time (RFS) was relatively short for patients with tumor TNM stage III and lymph node positive after radical resection. After adjusting for TNM staging, the expression of *CXCL1* (corrected *P* ≥ 0.925, corrected HR (95% CI) = 0.957 (0.38 - 2.409) was not significantly correlated with tumor-free survival in COAD patients. The patients with early TNM stage, good tumor differentiation, no tumor thrombus, lymph nodes (-), radical resection, and no tumor metastasis had a relatively long OS. After correcting factors as the TNM stage, the tumor differentiation, with or without tumor thrombus and performed radical resection or palliative operation, Multivariate COX regression model showed that the expression of *CXCL1* (corrected *P* ≥ 0.737, corrected HR (95% CI) = 0.898 (0.478 - 1.685) was not significantly correlated with OS. To further understand the relationship between the expression of *CXCL1* protein and prognosis in COAD patients, we carried out the stratified analysis. There was no perceivable correlation between the expression of *CXCL1* protein and RFS in the subgroup of clinicopathological factors. OS of CEA positive patients before operation was longer than that of *CXCL1* positive patients. (Corrected* P* = 0.005 corrected HR (95% CI) = 0.239 (0.087 - 0.656) (Fig 6).

#### Gene set enrichment analysis

**Figure 7:**
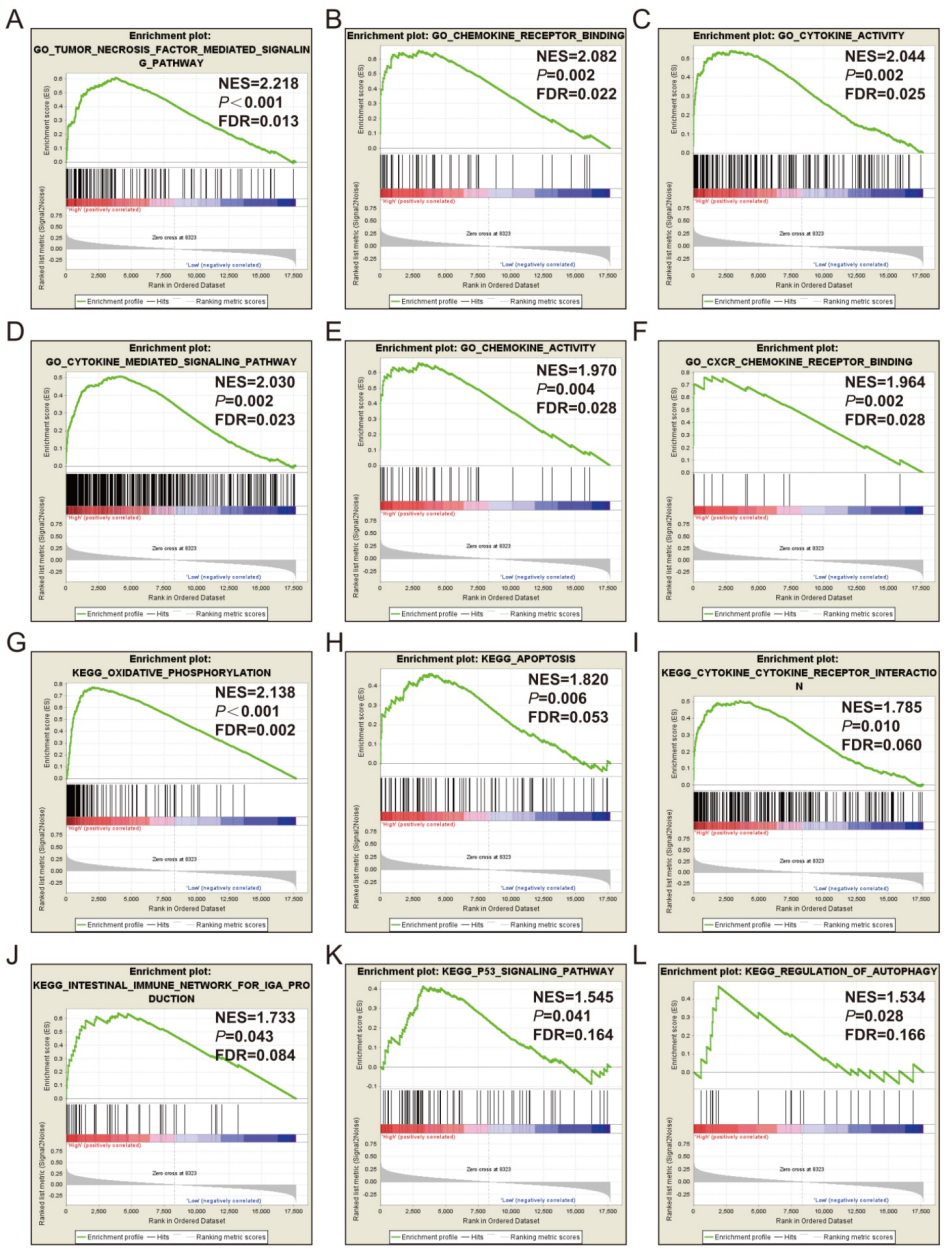
GSEA results of *CXCL1* expressed in COAD tumor tissues. Notes: (A-F) GSEA results of c5 reference gene sets for high *CXCL1* expression groups; (G-L) GSEA results of c2 reference gene sets for high *CXCL1* expression groups; ES, enrichment score.

GSEA of *CXCL1* was also performed by TCGA cohort. The RNA sequencing dataset of COAD patients was divided into 2 phenotypes through the median value of *CXCL1* expressions in tumor tissues. The results of GSEA were displayed in Fig. 7 and Table S, which indicated that the high expression of *CXCL1* was appreciably relevant to cytokine activity, cell apoptosis, *P53* regulation pathway and regulation of autophagy.

## Discussion

Cancer metastasis was still the main cause of death in CRC patients. The 5-year overall survival rate of CRC patients could be as high as 80-90%, but it would decrease to 5-10% after tumor metastasis 26, 27. Therefore, early detection of CRC are particularly important for patients' clinical outcome. Tumor markers with high sensitivity and specificity contributed to the early detection of tumors, and previous studies of CRC biomarkers had not yielded ideal results 28-33. In the prognostic study of CRC, some prognostic markers had been found to be used to screen the risk of recurrence or metastasis, however, their performance in clinical application was not perfect due to the limitation of technology, cost, and their complicated testing methods 34, 35.

In this study, by comparing the expression distribution of *CXCL1* in normal human organs and tissues, we observed that expression of *CXCL1* in intestinal tissues was higher than that in most other organs, indicating that *CXCL1* played an indispensable role in normal physiological process of intestinal tissues. At the same time, by comparing the expression of *CXCL1* between tumor and paracancerous tissues in COAD patients from TCGA cohort, we also observed that the expression of *CXCL1* was dysfunctional between tumor and paracancerous tissues, and *CXCL1* was significantly up-regulated in tumor tissues. We verified this result through the cohort of the first affiliated Hospital of Guangxi Medical University from the perspectives of genetic and protein levels. The diagnostic ROC curves also suggested that *CXCL1* had a high diagnostic value for COAD. These results were accord with Wen Y et al 11 and Zhuo C et al. 36.

In previous studies, multiple studies reported the prognostic value of CXCL1 in colorectal cancer 36-38, and there were reports verifying the molecular mechanism of CXCL1 in colorectal cancer through in vivo and in vitro experiments 39-43. In the TCGA cohort, Kaplan-Meier analysis showed that the OS of patients with high expression of *CXCL1* was longer than that of patients with low expression of *CXCL1*, and multivariate analysis showed a similar trend. In the Guangxi Medical University cohort, we found that the expression of *CXCL1* in tumor tissues was significantly correlated with preoperative CEA. In the sub-group of CEA positive, the OS of patients with high expression of *CXCL1* was longer than that of patients with low expression of *CXCL1*. This result was different from previous studies 36, 41, 44. Interleukin-8, *CXCL1,* and other chemokines had a strong chemotactic effect on a series of inflammatory cells, such as T cells, neutrophils, and basophils, but their entire functions had not been fully elucidated 45. Our study provided new evidence for the significance of *CXCL1* expression. The good prognostic effect of infiltrated *CXCL1* positive was most likely to indicate the immune function of this chemokine and the anti-tumor effect of inflammatory cells.

Through GSEA analysis, we enriched some meaningful biological functions and metabolic pathways. The research of Cabrero-de et al showed that chemokine CXC subfamily genes were widely related to the occurrence and development of CRC 38. Soreide K et al reported cell apoptosis was associated with the prognosis of CRC 46. There were also many studies reporting the correlation between *P53* and CRC 47-49. Zhou H et al.'s study suggested that autophagy was related to tumorigenesis and the protection of cancer 50. However, the role of autophagy in CRC remained unclear. The advantage of the present study compared with previous studies was that we used TCGA whole-genome RNA sequencing data and GSEA method to further investigate the molecular mechanism of CXCL1 in COAD.

Although we first found the diagnostic and prognostic value of *CXCL1* in COAD (rather than colorectal cancer), there were still some shortcomings in this study: a) There was imperfectness in the clinical information of COAD patients from TCGA database, and some important information such as tumor size, histological classification, degree of differentiation had not been provided. b) The sample size of this study was limited. c) Functional tests were needed to further verify the mechanism of the *CXCL1* gene in the occurrence and development of COAD.

## Conclusion

In this study, we found that the *CXCL1* gene might function as a potential biomarker for the diagnosis of COAD, and might serve as a prognostic biomarker for a specific subgroup of COAD. Investigation of the molecular mechanism of CXCL1 in COAD, GSEA revealed that *CXCL1* high expression phenotype was related to cytokine activity, cell apoptosis, *P53* regulation pathway, and regulation of autophagy. However, further research and verification were still needed in the future.

## Supplementary Material

Supplementary data.

## Figures and Tables

**Table 1:** Clinical factors sheet for COAD patients in TCGA cohort

Variables	Patients (n=438)	No. of event (%)	MST (days)	HR (95%CI)	Log-rank *P* value
Sex					0.545
Female	204	44(21.6%)	NA	1	
Male	234	54(23.1%)	2475	1.131(0.759-1.686)	
Age^&^					0.112
≤65	168	29(17.3%)	NA	1	
>65	268	68(25.4%)	2475	1.420(0.919-2.194)	
TNM stage^†^					<0.001
I	73	4(5.5%)	NA	1	
II	167	27(16.2%)	2821	2.240(0.781-6.421)	
III	126	31(24.6%)	NA	4.068(1.434-11.538)	
IV	61	31(50.8%)	858	11.291(3.980-32.026)	
*CXCL1*					0.045
	219	58	1881	1	
	219	40	2821	0.665(0.444-0.995)	

Note: ^&^Age information of 2 patients was not obtained; ^†^Tumor TNM staging information was not obtained in 11 patients; MST: median survival time; HR: hazard ratio; 95% CI: 95% confidence interval; NA: Not available.

**Table 2:** Correlation analysis between *CXCL1* expression and clinical characteristics in 212 patients with COAD

Variable			Negative	positive	χ^2^	P-value
Gender					3.720	0.054
	Male		20	117		
	Female		19	56		
Age (years)					0.087	0.768
	≤65		26	111		
	>65		13	62		
CEA^a^ (ng/ml)					6.671	0.010
	1-5		28	85		
	>5		10	83		
TNM stage					5.058	0.168
	I		9	21		
	II		8	50		
	III		14	78		
	IV		8	24		
Location					0.962	0.327
	Right		16	86		
	Left		23	87		
Tumor type^b^					0.214	0.898
	Invasive		2	9		
	ulcerative		28	125		
	mass		9	33		
Tumor differentiation					1.401	0.496
	Well		3	7		
	Moderately		27	133		
	poor		9	33		
Tumor trombus^c^					0.415	0.519
	No		33	152		
	Yes		6	20		
Tumor size^d^ (cm)					0.754	0.385
	<5		19	71		
	≥5		19	97		
Tumor number					<0.001	1.000
	Single		38	167		
	Two		1	6		
Lymph node^e^					0.086	0.769
	Negative		23	97		
	Positive		16	75		
Tumor transfer					0.890	0.346
	No		31	148		
	Yes		8	25		
Nerve infiltration^f^						0.561
	No		38	169		
	Yes		1	3		

Notes: a, 6 patients without CEA information; b, 6 patients without general classification information; c, 1 patient without tumor thrombosis information; d, 6 patients without tumor size information; e, 1 patient without lymph node information; f, 1 patient without nerve infiltration information; NA: Not available.

**Table 3:** Associations between clinical factors with OS and RFS in 212 COAD patients

Variable					OS*					RFS^#^	
			Patients	MST (days)	HR (95% CI)	Log-rank *P*		Patients	MRT (days)	HR (95% CI)	Log-rank *P*
Gender						0.801					0.467
	Male		137	NA	1			111	NA	1	
	Female		75	NA	0.934 (0.552-1.582)			64	NA	1.302 (0.637-2.661)	
Age (years)						0.536					0.915
	≤65		137	NA	1			109	NA	1	
	>65		75	NA	1.174 (0.707-1.950)			66	NA	1.040 (0.505-2.143)	
CEA (ng/ml)						0.169					0.462
	1-5		113	NA	1			101	NA	1	
	>5		93	NA	1.424 (0.858-2.363)			70	NA	0.759 (0.364-1.585)	
	Missing		6					4			
TNM stage						<0.0001					0.039
	I		29	NA	1			30		1	
	II		57	NA	0.742 (0.209-2.629)			58		0.499 (0.144-1.725)	
	III		89	NA	2.469 (0.864-7.060)			87		1.625 (0.612-4.311)	
	IV		27	405	22.253 (7.607-65.098)			0			
Location						0.806					0.627
	Right		102	NA	1			86	NA	1	
	Left+both		110	NA	0.929 (0.565-1.529)			89	NA	1.191 (0.587-2.418)	
Tumor type						0.691					0.358
	Invasive		11	NA	1			7	NA	1	
	ulcerative		153	NA	1.511 (0.367-6.221)			126	NA	25897.646 (0-2.4428763792086E+116)	
	mass		42	NA	1.203 (0.267-5.428)			36	NA	17555.202 (0-1.657925218057E+116)	
	Missing		6					6			
Tumor differentiation						0.019					0.639
	Well		10	NA	1			10	NA	1	
	Moderate		160	NA	1.451 (0.352-5.993)			138	NA	0.582 (0.175-1.941)	
	Poor		42	NA	3.076 (0.710-13.318)			27	NA	0.724 (0.173-3.031)	
Tumor thrombus						<0.0001					0.095
	No		185	NA	1			163	NA	1	
	Yes		26	660	4.571 (2.568-8.134)			12	NA	2.389 (0.833-6.858)	
	Missing		1								
Tumor size (cm)						0.236					0.573
	<5		90	NA	1			75	NA	1	
	≥5		116	NA	0.739 (0.447-1.221)			95	NA	0.817 (0.404-1.654)	
	Missing		6					5			
Tumor number						0.138					0.160
	Single		205	NA	1			170	NA	1	
	Two		7	1917	2.119 (0.768-5.844)			5	1900	2.687 (0.64-11.291)	
Lymph node						<0.0001					0.001
	Negative		120	NA	1			107	NA	1	
	Positive		91	NA	3.546 (2.075-6.061)			68	NA	3.411 (1.633-7.126)	
	Missing		1								
Radical resection						<0.0001					
	Yes		175	NA	1						
	No		37	481	11.536 (6.836-19.469)						
Tumor transfer						<0.0001					
	No		179	NA	1						
	Yes		33	401	14.344 (8.376-24.565)						
Nerve infiltration						0.173					0.152
	No		207	NA	1			173	NA	1	
	Yes		4	1079	2.572 (0.628-10.540)			2	412	3.864 (0.525-28.419)	
	Missing		1								
Postopetative chemotherapy						0.833					0.744
	No		69	NA	1			59	NA	1	
	Yes		124	NA	1.061 (0.610-1.846)			100	NA	0.887 (0.43-1.828)	
	Missing		19					16			
*CXCL1*						0.444					0.738
	Negative		36	NA	1			31	NA	1	
	Positive		166	NA	0.788 (0.427-1.452)			144	NA	0.859 (0.352-2.094)	

Notes: * 202 patients had OS information; # 175 patients underwent radical surgery, of which 171 had RFS information; MST: median total survival; MRT: median recurrence time, NA: Not available.
